# Immune Privilege Furnishes a Niche for Latent Infection

**DOI:** 10.3389/fopht.2022.869046

**Published:** 2022-03-08

**Authors:** John V. Forrester, Christine Mölzer, Lucia Kuffova

**Affiliations:** ^1^ Ocular Immunology Group, Section of Infection and Immunity, Institute of Medical Sciences, University of Aberdeen, Aberdeen, United Kingdom; ^2^ Eye Clinic, Aberdeen Royal Infirmary, Aberdeen, United Kingdom

**Keywords:** uveitis, retina, CNS, barrier, microbe, tolerance, intraocular inflammation, latency

## Abstract

The microenvironment of the CNS (eye and brain) is fertile ground for infection if the barriers are breached. The result of pathogen invasion is often devastating destruction of tissues. In the eye, inflammation is broadly classified either as “infectious” (i.e. caused by infection) or “non-infectious”. However, increasingly, forms of intraocular inflammation (IOI), which clinically appear to be “non-infectious” turn out to be initiated by infectious agents, suggesting that pathogens have been retained in latent or persistent form within ocular tissues and have reactivated to cause overt disease. A similar pathogenesis applies to latent infections in the brain. Not all CNS tissues provide an equally protective niche while different pathogens escape detection using different strategies. This review summarises how immune privilege (IP) in the CNS may be permissive for latent infection and allow the eye and the brain to act as a reservoir of pathogens which often remain undetected for the lifetime of the host but in states of immune deficiency may be activated to cause sight- and life-threatening inflammation.

## Introduction

The host-pathogen interaction is not an easy one. Either the host or the pathogen fails to survive. A good outcome for the host is one in which the pathogen is completely cleared. Whether this is achieved depends on the fitness of the host and the potential for the pathogen to wreak damage (virulence). Less virulent pathogens have evolved strategies to evade the host’s immune defences, and these are variously described as latency, dormancy, persistence, or immune evasion. This ensures species survival for the pathogen assisted by a conducive host environment.

Hosts are generally complex organisms comprised of multiple parts (tissues) each with varying levels of host defence capability, an immunological feature described by Matzinger as “tissue-based” control of the immune response (1). This may be further modulated in chronic/persistent infection in that the infected tissue might alter host immune cell behaviour, for instance towards effector memory T cell exhaustion (2) or increased T regulatory [Treg] cell activity (3, 4). Central nervous system (CNS) tissue (eye and brain) is known to modify the immune response, a property attributed to its “privileged” status (immune privilege, IP). Although all tissues exhibit IP to varying degrees, in the eye and the brain IP’s immunomodulatory properties are maximised (5). This review considers the possibility that the IP status of the CNS offers pathogens special license to evade the immune system, often for the lifetime of the host, by promoting latency.

## Immune Privilege Is One Form of Immunological Tolerance

The concept of IP developed as an explanation for a phenomenon that did not fit with the emerging dogma on immunological tolerance (IT). IT, as a theory, developed from studies of allograft rejection and the discovery of MHC antigens, and was formulated on the notion of self-non-self-discrimination (SNSD) (6). Immunity to foreign antigens was viewed predominantly in terms of the specificity of adaptive immune responses and SNSD-based IT was the process whereby the immune system avoided T and B cells turning on the host.

SNSD-based IT is described mechanistically as central (thymus-based) or peripheral, both of which occur mainly through three mechanisms: deletion, anergy or regulation (T, B reg) of self-reactive effector T and B cells [reviewed in (7, 8)]. However, the SNSD paradigm did not appear to apply to the eye where (non-self) skin allografts placed in the anterior chamber of the eye were accepted despite rejection of the same allograft in the skin (9). Accordingly, IP was coined as a term to describe this unique property of the CNS (similar allografts in the brain were accepted) and was extended to include foreign antigens generally [reviewed in (10)] although this has been debated with regard to infectious agents (11–13).

The original SNSD theory did not apply to the primary (innate) immune defence system which was considered to respond non-specifically to foreign antigens. However, when innate immune cells were found to display selectivity in their responses to different classes of pathogens and toxins (pathogen- or damage-associated molecular patterns: PAMPs, DAMPs) the role of innate immune cells as drivers of adaptive immunity became clear (14, 15). Thus the “Danger” or “Damage” model was formulated in which the immune system responds to pathogenic stimuli through innate immune cell recognition of PAMPs/DAMPs by pattern recognition receptors (PRR) and presents the engulfed and processed antigens to adaptive immune cells. In addition, activated T and B cells not only clear pathogens (e.g. CD8 cytotoxic T cell killing of virus-infected cells) but also generate antigen-specific memory cells (16). All antigens, including self-antigens, can thus potentially activate the immune system but only do so in the right context (co-stimulation) and microenvironment.

Immune responses are therefore highly context-dependent and the role of the tissues as the site of host-pathogen encounter is also recognised (1). Under normal circumstances, the overwhelming majority of antigens (foreign and self) do not induce a clinically detectable tissue-damaging immune response, as evidenced by the enormous range of harmless commensal antigens, plus the potential innumerable self-antigens, and if they do so, the tissue has the capability to modify the outcome and restore homeostasis (17). However, host-microbe interactions are not binary in their responses since several outcomes are possible. From a conceptual point of view, the host-microbe relationship has thus been re-branded as the damage-response framework (DRF) to encapsulate the range of interactions and includes five existential microbial states within a host: infection, colonisation, commensalism, disease and latency (18).

When immune responses in the eye are considered in this context, it offers an explanation for IP: the microenvironment of the eye is not conducive to “conventional” immune responses as occur in the skin and achieves this state using the same mechanisms as IT, namely deletion [e.g. Fas/FasL, TRAIL/TRAIL-Rs (1–4), CTLA-2α/PDL-1 [ (19–22)], anergy (23, 24) and T/Breg, controlling both innate and adaptive immune responses (25–27). Robust, conventional immune responses, for instance in the lung, the gut or the skin, have a good chance of clearing the infection and restoring tissue homeostasis in an otherwise healthy individual. In contrast, the attenuated immunity of IP does not seem to be very effective either in clearing the infection or preventing intraocular inflammation (IOI) [uveitis is a significant cause of blindness, www.who.int/blindness (28)]. Moreover, IP has not been demonstrated convincingly for infectious foreign antigens [reviewed in (5)]. In fact, when infectious agents invade the eye the extent of tissue damage can be catastrophic. But this is not always the case; some cases of intraocular inflammation caused by infectious agents [such as toxoplasma-associated uveitis (29)] can resolve. Furthermore, apparent non-infectious causes of intraocular inflammation are just about as frequent as infectious causes. How is this explained?

## Intraocular Inflammation

### Aetiology

Intraocular inflammation is a rag bag of conditions, described often by their clinical presentation but also by the causative agent. Etiologically, IOI is described as infectious when an infectious agent can be detected, isolated, and/or cultured from the intraocular compartment/tissues and is seen to be the cause of tissue damage. Molecular diagnostics are increasingly helpful in detecting microbes (30). “Infectious” in general terms implies transmissibility but this does not occur in IOI unless the ocular surface and the surrounding adnexae are involved as in herpes zoster ophthalmicus (31) and SARS-CoV-2 conjunctivitis (32). For instance, cataract surgery in survivors of the highly infectious Ebola virus (33) did not lead to transmission of disease even though they had recent signs of IOI (34). Rarely, infectious agents can be transmitted *via* corneal allografts in unsuspected donor infection [e.g. Chikungunya (35), herpes simplex (36), and prion disease (37)] but this does not come under the typical classification of “infectious” IOI. In contrast, non-infectious IOI is the label for conditions in which no infectious agent can be detected.

### Clinical Presentations

The Standardisation of Uveitis Nomenclature (SUN) working group has sought to bring order to the descriptors for the range of syndromes collected under the umbrella of IOI, and defined sets of clinical criteria have been proposed for each condition mainly to facilitate a bioinformatic approach to clinical studies (38). The initial SUN criteria are based on anatomical descriptions relating to the location of the primary site of inflammation in the eye (39). The more recent subset classification has necessarily been more restrictive, and some cases of IOI will not fit the criteria for any specific clinical entity. However, the overarching concept retains the possibility that some individual conditions can present in a variety of ways such as tuberculous or syphilitic IOI and that pathogenetic tissue-damaging processes are common to many of the conditions. This is important to realise since the level of inflammation and damage varies with the tissue involved and its intrinsic degree of tissue tolerance (IP) (5).

### Privileged Tissues Involved

We, and others (40–42), have argued that IP is relative: some tissues exert greater control over immune responses compared to others and that this applies particularly to CNS tissues. Thus, the retina and the brain parenchyma have a range of physical, chemical and immunological barriers which provide a high level of IP while border tissues such as the uveal tract and the meninges, with their rich complement of immune cells and ready access to draining lymph nodes have low levels of IP. The brain parenchyma (and presumably retina) in fact, have connections with the secondary lymphoid tissues (cervical lymph nodes) through drainage of interstitial fluids and cerebrospinal fluid (CSF) *via* the recently recognised brain glymphatic system and meningeal lymphatics (5, 10, 41). Tissues such as the cornea and sclera have intermediate levels of IP, mainly due to their low vascularity and limited expression of MHC molecules (43) as well as their content of immunomodulatory mediators (21). Indeed, corneal IP is readily rescinded by infection (44).

## Latent Infection as a Cause of Infectious Uveitis

Microbial latency is described operationally as a state “in which host damage that occurs does not perturb homeostasis to a degree that results in clinical disease” (45). An important difference between colonisation and latency is time: colonising microbes are eventually cleared or cause overt disease while latent microbes usually persist for the lifetime of the host, frequently without causing disease as for instance with Epstein Barr virus (EBV).

Latent infections can be caused by a wide range of microorganisms and occur in many sites. They have a predilection for residency in the CNS, either in parenchymal tissues per se or in the brain and eye border regions (meninges and uveal tract) (10). The following sections illustrate various forms of microbial latency in the CNS and eye.

### Fungi

Several species of fungi can cause damaging inflammation in the eye and brain including *Cryptococcus neoformans, Aspergillus* spp.*, Histoplasma capsulatum*, and *Fusarium* sp. Fungi are typically not dependent for replication on a mammalian host and usually survive and replicate environmentally. While many infections are exogenous, some are endogenous and occur only when the host is immunosuppressed, suggesting that the microbe is reactivated from a “dormant” state after subclinical invasion. Fungi require two conditions to establish overt tissue-damaging infection: dormancy and immunosuppression (46). *Cryptococcus neoformans* infection is contracted early in life and persists in myeloid cells in granulomas in the lung and draining lymph nodes asymptomatically. How they are released from infected lymph nodes to enter the circulation is poorly understood but they invade the CNS when they have passed from the circulation into the CNS border regions (uveal tract and meninges). Access to the retina and brain is usually *via* direct passage across the BBB/BRB (transcytosis) or more commonly, inside migratory leukocytes (Trojan Horse mechanism) (47).

How fungi achieve a state of dormancy (a state of greatly slowed but still functioning metabolism) varies somewhat with each organism. For instance, *C. neoformans* may be inhaled either as yeast or a spore, but while yeasts are killed after phagocytosis by lung macrophages, spores only weakly stimulate PRRs and so can persist for long periods of time in macrophages and dendritic cells within granulomas (48). Granuloma formation is an important mechanism in controlling *C. neoformans* and involves IFNγ**−** producing Th1 cells within the lesion. Yeast glucuronoxylomannan (GXM, 80%) and galactoxylomannan (GalXM, 10%) are the major capsular polysaccharides which influence the immune responses (49). As for the lung, it is likely that *C. neoformans*-containing uveal and meningeal granulomas persist for long periods undetected (latent infections) until protective immunity (~CD4/CD8/NK cell) declines to a point where it fails to prevent extrusion of replicating fungi from macrophages and entry into the CNS parenchyma. The fungus may also contribute to this process since GXM, shed from yeast has immunosuppressive properties, reducing immune cell entry into the CNS (50). In addition, fungal urease seems to be required for invasion of the CNS [reviewed in ref (51)].

Other fungi which cause endogenous CNS infections, such as *Aspergillus*, may follow a similar pattern of pathogenicity of latency/dormancy and disease when immune control declines (52). They may even invade the CNS locally along nerve endings from the paranasal sinuses through cribriform plate (53–56) a route also taken by some viruses such as Ebola and SARS-CoV-2 COVID -19 (see below). *Pneumocystis carinii* occurs in normal lung tissue and does not usually cause pneumonia unless the patient is severely immunocompromised (57, 58). Spread to the choroid can then occur to cause disease. Whether it can reside latently in choroidal macrophages is not known. However, fungi generally such as *Candida* (the most common fungal infection), *Fusarium* and *Mucorales* probably do not fall into the category of latent infections, unless they have progressed through a barrier-breaching invasive phase of infection e.g. in the lungs or gut, and have reached a second stage of immune evasion by persistence as dormant microbes within myeloid cells, controlled by an adaptive immune response. In the absence of this process, they are better considered to be commensals, symbionts or parasitic-but-potentially-infectious agents as defined by the DRF (18).

### Parasites/Nematodes/Helminths

*Toxoplasma gondii* (Tg) is the most common parasitic infection of the eye and in some regions such as Brazil is the commonest cause of IOI (59). Infection is acquired at any age mostly through ingestion of infected meat. Serological evidence of exposure to Tg increases with age and in some countries can reach levels of 80-90% of the population. However, most individuals are asymptomatic, or experience only a mild GI disturbance (60, 61).

Ingestion of Tg cysts (bradyzoites/oocysts) leads to the release of large numbers of tachyzoites on contact with digestive enzymes which rapidly invade the blood and lymphatic circulations. Tachyzoites disseminate freely or inside infected dendritic cells induced by the Tg 14-3-3 protein (62), and generate a marked IFNγ Th1 response in draining lymph nodes. Both free or leukocyte-internalised tachyzoites invade muscle tissue and cross the BRB/BBB to infect neurons where they are induced to transform to bradyzoites *via* formation of a parasitophorous vesicle. In this state they are considered to have entered a prolonged phase of latency, but in fact there is a low level of Tg replication within bradyzoites (63). Replication is inhibited by the continuous production of antigen-specific CD8 T cells which require CD4 T cells to “help” their generation (64). Reactivation of latent CNS Tg thus depends on failure of both CD4 and CD8 T cell responses (65). In addition, recent studies in Balb/c mice have shown that a specific peptide from Tg, Gra6, binds an unusual MHC Class I molecule, MHC-1 L(d), to enhance CD8 T cell protection against Tg reactivation (66, 67). Other cells are also involved in this CNS protective response: astrocytes and oligodendrocytes release IL33 in Tg infected brains (66) and probably also in Tg infected eyes (68), and is required for control of infection *via* chemokine-induced recruitment of immune cells, while microglial production of gasdermin adds a further layer of immune protection (69). Thus, the complexity of the immunological machinery required to keep Tg bradyzoites in latency is critically dependent on the CNS microenvironment.

Other latent parasitic infections affecting the eye are unusual. Malaria, giardiasis, leishmaniasis, and trypanosomiasis have all been reported, and ocular disease occurs often long after the initial infection, raising the possibility of an initial asymptomatic infection and sequestration of parasites in the eye. For instance, it is estimated that up to 33% of patients with giardiasis develop late extraintestinal manifestations including ocular disease (often in the form of retinal vascular occlusion and/or uveitis) and arthritis [reviewed in ref (70).]. The pigmentary degeneration which is characteristic of late ocular disease suggests that the parasite may reside latently in the retinal pigment epithelium (RPE) (71, 72).

Numerous other parasites, nematodes, cestodes and trematodes, as well as helminths are known to cause ocular infections [reviewed in ref (73)], but most would not be classified as latent infections. However, they often induce minimal inflammation as in the case of intraocular loiasis (74) or in cases of DUSN (75) prior to onset of symptoms, and the invading parasites (e.g. Loa loa) might be regarded as having a symbiotic, mutualistic or even a commensal relationship with the intraocular compartment. In another tissue, such parasites would likely induce a vigorous immune response and be rapidly cleared by the immune system.

### Viruses

A large number of viruses, both DNA and RNA, are recognised to establish latent CNS infections and cause CNS inflammation and IOI when reactivated. Top of the list are herpes viruses including HSV-1 and HSV-2, VZV, CMV and EBV. Herpes viruses have tropism for particular cells (neurons: HSV, VZV; myeloid cells: CMV; B cells: EBV; T cells: HIV) and reside in CNS cells or in leukocytes populating the CNS border regions (uvea, meninges). They infect neighbouring cells by shedding virus-containing exosomes (76). CMV establishes latency in haemopoietic cells and renders them inactive/anergic (77) thereby promoting latency (78). Reactivation of most DNA viruses occurs in immunosuppressed or immunodeficient patients indicating that latency and control of reactivation is actively mediated by the immune system. Interestingly, as for parasites, control is achieved through CD8 T cell activity (79).

EBV has been implicated in the pathogenesis of multiple sclerosis (MS) for many years. Despite evidence of EBV in the brains of MS patients (80), resistance to this concept has been long standing (81, 82) in part because EBV infection is so ubiquitous [EBV seropositivity is present in over ~85% of the population (83)]. However, a recent extensive study in US military personnel of the relationship between EBV antibody seroconversion and newly onset MS strongly suggests that EBV is required as a trigger for MS disease (84), and that certain specific proteins (EBER, EBNA-1) act as molecular mimics of CNS proteins such as glial CAM (85) and anoctamin 2 (86). As indicated above, EBV latently infects B cells, which account for ~20% of immune cells in the immune cell rich meninges. MS patients consistently develop B cell granulomas in the meninges which extend into the subarachnoid space and pial meninges lining the cortex where the inflammatory damage and virus spreads to glial cells and oligodendrocytes, eventually affecting axonal transport and neuronal function (87–89). Reactivation of EBV *in situ* would thus cause extensive damage at the cortical level.

RNA viruses such as Ebola, Chikungunya (90), Rubella (91), Dengue (92), Measles (93), Zika (94) and more recently SARS-CoV-2 are known to persist in the CNS and are recognised causes of IOI in survivors from the primary infection (95). Here, the definition of latency is less clear. Current thinking is that such viruses develop persistence (i.e. low-level replication) rather than latency, as in the case of Ebola which persists as a slowly replicating virus in the RPE (96). Even with vaccination, Ebola can persist and reactivate with dire consequences (97).

Latent HIV persists in CNS microglial reservoirs (98) and can cause retinopathy. CNS HIV persistence can impinge negatively on efficacy of treatment (99). The retrovirus HTLV-1, a world-wide cause of IOI (100, 101), reactivates when the immunoregulatory aryl hydrocarbon receptor (Ahr) (102) IDO system is functional: reactivation is controlled by the level of Ahr ligands and balanced by persistent activation of NF-кB (103). Thus, this immunoregulatory system, which is active within the immune-privileged eye (104, 105), may determine whether HTLV-1 induces uveitis. Like Ebola and RNA viruses generally, HTLV-1 persistence is maintained by low level replication and in the CNS microenvironment is at risk of reactivation. As for most viral infections, HTLV-1 is a “life-long infection which is never truly silent” (106).

Interestingly, “subclinical” virus reactivation in the form of viral shedding (for instance in the urine) is a common phenomenon and is exacerbated by many forms of stress (107).

### Bacteria

**Figure 1:**
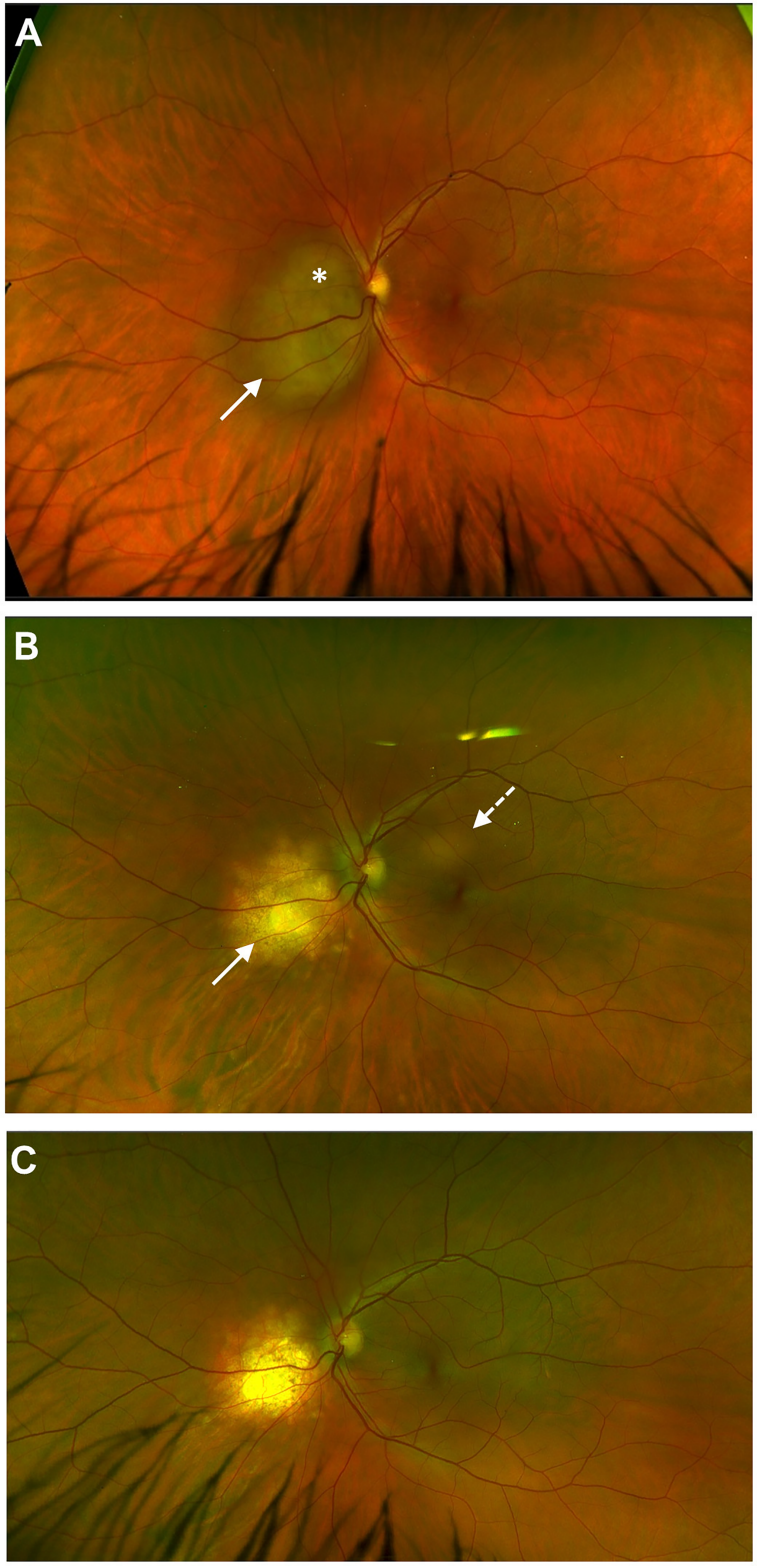
Choroidal tuberculoma* in HIV +ve patient two years after cessation of anti-retroviral therapy. Miliary tuberculosis involving eye **(A)**, CNS, multiple lymph nodes (mediastinum, retroperitoneal) and spleen; regression of initial lesion (white arrow) but appearance of a new lesion (dashed arrow) after six months of anti-TB and anti-retroviral therapy **(B)**; anti-TB therapy stopped at 12 months; complete resolution of both lesions noted at 18 months **(C)**.

*Mycobacterium tuberculosis* (Mtb) is the paradigmatic cause of latent bacterial infection involving the CNS. The disease is contracted as an airborne lung infection and produces two forms: caseating tuberculosis when the organism is replicating and causing tissue necrosis, and immunoreactive TB where replicating Mtb is held in check by a prominent immune response. The latter takes the form of a granuloma, either in the lung or in the draining lymph node (108) and, as for many microbes, the organism evades direct attack by the immune system by residing in myeloid cells (macrophages and DC) where it disables the lysosomal killing machine (109). Viral co-infection promotes survival of Mtb in macrophages by co-opting TNFα to reduce T cell priming (110). However, intracellular Mtb proliferation is controlled by a CD4 T cell response but this is a complex interaction between the host and the pathogen and this immune response may be either protective or pathogenic (tissue damaging) (111). Infected myeloid cells, or, in the absence of CD169, their shed extracellular vesicles (112), escape the granuloma and disseminate to cause extrapulmonary TB (miliary TB) with a predilection for CNS border tissues. This presents as TB meningitis and choroiditis and is typically contained as a granulomatous immunoreactive inflammation. However, when immune dysregulation looms, caseation and tissue necrosis within a “tuberculoma” can occur (**Figure 1**). Ocular TB is a classic example of latent infection safely sequestered in macrophages: latent TB IOI disease in its miliary form does not contain replicating bacilli while the choroidal tuberculoma may contain replicating microbes and be “infectious” in the true sense.

TB is widespread: one third of the world’s population have been infected with TB, most of whom survive the disease and present with latent TB, demonstrated by a positive skin test (TST, Mantoux) and/or interferon gamma release assay positivity (Quantiferon test). These tests indicate that the patient has been prior infected by Mtb and probably that the tubercle *Bacillus* is resident in the host with the capability to induce pathology even though the lung may have cleared the infection (113). Latent TB is long-lasting and clearance of Mtb is probably never complete (113, 114). Disease reactivation involving the CNS parenchyma occurs particularly in states of relative immunosuppression but is more commonly restricted to the CNS border regions. Despite large numbers of latently infected people, <10% develop overt disease: this suggests that persistence of latent Mtb is the norm in immunocompetent individuals and that the site of latency is frequently the CNS border regions (meninges and uveal tract).

Other latent bacterial infections in the eye are uncommon. *Treponema pallidum* becomes “latent” (i.e. clinically silent) after resolution of the primary and secondary stages and manifests itself in the tertiary stage in the CNS and eye. During latency, control of spirochete replication is maintained by a robust immune response such that progression from the latent stage to the tertiary stage is not inevitable, occurring in around 15-40% of cases (115). Patients in the latent stage who do not progress may have cleared the infection (with the help of antibiotic therapy) but may also harbour non-replicating spirochetes. Interestingly, the spirochete traverses the blood-CSF barrier without causing neurological disease in the early stages (116), resides in the CNS border regions during the latent period (117) and can be detected by CSF PCR technology (118, 119).The same occurs with *Borrelia Burgdorferi*, the tick-borne etiologic agent of Lyme disease and a rare cause of IOI (120). Another tick-borne disease, Rickettsia, has been reported to cause “epidemic rickettsia retinitis”. Such cases develop as a later event after the primary infection and are diagnosed by the serological Felix-Weil test (121). However, such cases probably do not fit the diagnosis of latent infection. *Bartonella ssp.*, a further vector borne pathogen can cause IOI in rare cases, and can present in various forms including serpiginous choroiditis (122). Lastly, certain cell wall deficient and mycoplasma-like organisms have been reported in chronic intermediate uveitis, but their nature is obscure (123, 124).

## Latent Infection as a Cause of Non-Infectious Uveitis

A recent study based on insurance claims indicates that <20% of cases of uveitis in the US are directly caused by infection (125). In developing countries, the numbers are higher but accurate statistics are difficult to obtain. The conundrum is what causes IOI when no infectious agent can be identified [“non-infectious” or “undifferentiated” uveitis (126)] even with cutting edge molecular diagnostics (127)? Autoimmunity as a cause has fallen from favour mainly due to the lack of evidence for pathogenetic autoantibodies or T cells different in quality or quantity from those that occur in healthy populations. Similarly, evidence for autoinflammatory mechanisms in IOI is mostly restricted to rare monogenetic syndromes although dysregulated inflammation is gaining traction as an underlying predisposition [reviewed in (126)]. The possibility that infection, either directly or indirectly, causes most if not all forms of IOI and that its aetiology is only masked by the limitation of the diagnostic toolkit warrants consideration. The following paragraphs address some clinical IOI entities.

### Intermediate Uveitis

Intermediate uveitis includes the subgroups pars planitis, vitritis, and the peripheral retinal vasculitis of MS. The retinal parenchyma is not generally involved. All of the above classes of microbes have been associated with intermediate uveitis (90, 128–132). Latent tuberculosis has been implicated in peripheral occlusive retinal vasculitis (previously known as Eales’ disease) (133–135), while latent EBV infection is a strong candidate for the same pathology in MS (84, 136). Pars planitis with or without snowbanking has been linked to parasitic infections such as toxocariasis (137) while vitritis has long been associated with low level infections with *mycoplasma* and other cell wall deficient bacteria (138, 139).

### Anterior Uveitis

Increasingly, viral aetiologies have been identified for many cases of chronic, recurrent uveitis including rubella, VZV, CMV and HSV-1 and HSV-2. Patients recovering from Ebola and Zika virus infection develop recurrent/chronic anterior uveitis (see above section on viruses). *M. tuberculosis* and sarcoidosis are known causes of granulomatous uveitis; while Mtb has not be definitively identified as the cause of sarcoidosis, the disease has been linked to atypical mycobacterial infection (140). On a different note, HLA B27-associated acute anterior uveitis is associated with a disturbed microbiome in which translocating gut commensals have been incriminated (141, 142).

### Retinitis

Retinitis can be caused by all classes of microorganism described above. However, the AIDS epidemic brought viral infections as a cause of retinitis to attention particularly CMV, HSV-1 and HSV-2 and VZV. Exposure to these viruses is widespread in the population (see above section on viruses). Initial infection occurs early in life and latent infection is controlled by the immune system for the lifetime of the host. CMV resides in myeloid cells in the uveal tract and can pass to RPE cells by direct spread. CD8+ T cells control latent virus which replicates slowly and spreads contiguously in a brush-fire or patchy fashion when CD4/CD8 T cell immunity is impaired (143).

### Choroiditis

Choroiditis as a discrete entity is uncommon and takes several clinical forms including multifocal choroiditis and serpiginous choroiditis. Tuberculosis is strongly implicated in serpiginous disease (144) while several infectious aetiologies have been linked to cases of acute posterior multifocal placoid pigment epitheliopathy (APMPPE) (145–153).

## Are Most Forms of Intraocular Inflammation Infectious?

Although in developed countries, a microbial aetiology for non-infectious uveitis is often elusive, the door to an infectious aetiology has been left open by the re-branding of “non-infectious” IOI to “undifferentiated” IOI (38). In effect, this terminology admits that cases of IOI/uveitis, in which a microbial aetiology cannot be identified, also cannot be differentiated clinically from cases of infectious IOI, and despite the range of clinical presentations the commonality between infectious and “non-infectious” is striking.

As detailed above, pathogenetically, infectious agents initiate disease by entry across mucosal or skin barriers in which the primary infection may or may not be symptomatic. If the initial infection is not completely cleared, in line with the DRF concepts, it is likely that one of the five potential outcomes of this initial interaction occurs: infection, colonisation, commensalism, disease and latency. Both disease and latency outcomes require systemic microbial dissemination and localisation to the target site. For many of the clinical conditions described above, reactivation from latent infection is the most likely pathogenetic explanation since latency is a function of both the CNS microenvironment and a robust immune response. It is this immune response which promotes latency *via* both CD4+ and CD8+ T cell activity for the lifetime of the host. Only when immunity declines (immunosuppression, AIDS, aging) does the latent microbe reactivate and replicate causing, in the case of the eye, devastating IOI. We argue therefore that most if not all cases of IOI are caused by, and are the consequence of, a host-microbe interaction and the nature of tissue damage varies dependent upon whether the microbe is replicating and causing direct tissue necrosis (e.g. CMV retinitis) or whether there is an exaggerated immune reaction to the pathogen as occurs in reactivated Ebola and Mtb latent uveitis.

## Conclusion

**Figure 2:**
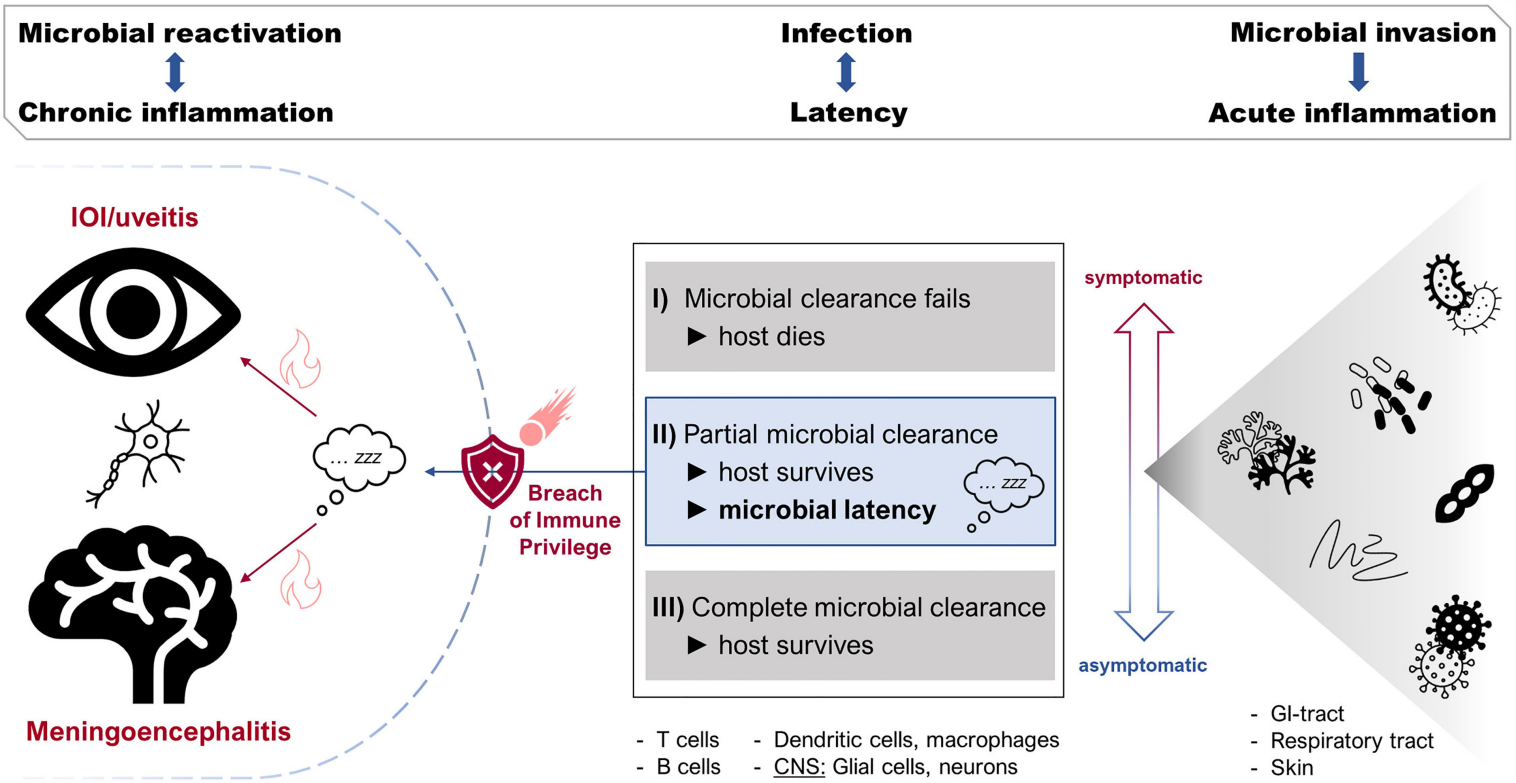
Host microbe interaction: the precarious relationship between immune privilege (IP) and latent infection. Microbial challenge and invasion of the host through external barriers (cartoon on right) causes acute inflammation and spread of infection with one of three outcomes (box in centre). The host survives if the infection is fully cleared or if the infection becomes latent. Keeping microbes in a latent state depends not only on a conducive (“privileged”) microenvironment such as that in the CNS but on a sustained immune response. The microbe can reactivate when IP is breached by immune dysregulation (cartoon on left).

The CNS acts as a preferred site for latent infections, not only through its complement of stable, quiescent structural cells, its physico-chemical and its immunological barriers, but also in providing a microenvironment where immune cells can reside in a “housekeeping”, homeostatic role i.e. the meninges and the uveal tract. This allows parasitic, bacterial, fungal and viral infections, disseminating from sites of infection elsewhere, to set up home as latent infections within immune cells (T cells, B cells, and macrophages, dendritic cells, and microglial cells) as well as parenchymal cells, which act as safe havens for microbes to evade the immune system (154) (**Figure 2**).

Inflammation generally is now considered to be an integral part of many diseases, previously considered to be genetic, metabolic, or degenerative (155). The driver for this “physiological” inflammation is likely to be microbial and related to the microbiome since the microbiome is central to the development of both the innate and the adaptive immune response (156). Certain unconventional T cells in the gut and elsewhere (iNKT cells, γδ cells and MAIT cells) imprinted in neonatal life are critical for tissue homeostasis (157) and many of the infections discussed here are contracted in early childhood.

When the system is perturbed as occurs on pathogen challenge, the organism acts to clear the pathogen and restore homeostasis, achieved through inflammation (17). This may be completely silent (asymptomatic infection) or life-threatening, but if the host survives, its clearance of the pathogen is unlikely to be complete, and persistent (latent) pathogens in the host continue to drive the homeostatic (immunogenic) response. At times, this may take the form of overt inflammation (IOI) or may be silent, and the eye is an especially sensitive sensor of this response as for instance with the “pepper-and-salt” pigmentary retinopathy of congenital rubella or childhood giardiasis. It is no surprise therefore that “undifferentiated” IOI is similar to infectious IOI and, whether the pathogen is detectable or not, it is a sobering thought that many of us, having been exposed to these pathogens, continue to harbour them within our cells and tissues in apparent good health until, that is, our immune systems let us down.

## Author Contributions

JVF conceptualised and wrote the article. CM created figures. LK provided images. All authors critically revised and edited the manuscript and provided expert input. All authors contributed to the article and approved the submitted version.

## Funding

University of Aberdeen Development Trust/Saving Sight in Grampian: grant number RG16220-10

## Conflict of Interest

The authors declare that the research was conducted in the absence of any commercial or financial relationships that could be construed as a potential conflict of interest.

## Publisher’s Note

All claims expressed in this article are solely those of the authors and do not necessarily represent those of their affiliated organizations, or those of the publisher, the editors and the reviewers. Any product that may be evaluated in this article, or claim that may be made by its manufacturer, is not guaranteed or endorsed by the publisher.
